# Photon‐Sphere Modes in Curved Optical Microcavities: A Black‐Hole Analogue Laser

**DOI:** 10.1002/advs.202517466

**Published:** 2026-03-06

**Authors:** Chenni Xu, Aswathy Sundaresan, Nazire‐Begüm Kazkal, Clement Lafargue, Lior Zarfaty, Li‐Gang Wang, Ofek Birnholtz, Dominique Decanini, Melanie Lebental, Patrick Sebbah

**Affiliations:** ^1^ Department of Physics, The Jack and Pearl Resnick Institute for Advanced Technology Bar‐Ilan University Ramat‐Gan Israel; ^2^ Université Paris‐Saclay, CNRS, Centre de Nanosciences et de Nanotechnologies Palaiseau France; ^3^ Laboratoire Lumière, Matière et Interfaces (LuMIn) CNRS, ENS Paris‐Saclay Université Paris‐Saclay, CentraleSupélec Gif‐sur‐Yvette France; ^4^ School of Physics Zhejiang University Hangzhou China

**Keywords:** astrophysical phenomena emulation, curvature potential trapping, curved microlasers, non‐Euclidean photonics, quasinormal modes

## Abstract

The bell‐like ringdown of the gravitational field in the final stage of massive black‐hole mergers is now routinely detected on Earth by the latest generation of gravitational‐wave detectors. Its spectrum is interpreted as a sum of damped sinusoidal vibrations of spacetime in the vicinity of the black hole. These so‐called quasinormal modes are the subject of extensive current studies, yet their physical nature remains elusive. Here, we emulate in the laboratory genuine four‐dimensional (3+1)D black‐hole metrics using an effective (2+1)D optical metric defined on a two‐dimensional curved surface that preserves the features of light‐like geodesics. We analytically compute the quasinormal modes of the optical cavity and show that, in addition to conventional whispering‐gallery modes (WGMs) supported near the cavity boundary, a new family of modes is confined around the photon sphere, the unstable region where spacetime curvature traps light in circular orbits. By 3D‐printing non‐Euclidean dye‐doped microcavities, we demonstrate lasing in both WGMs and photon‐sphere modes, with the latter exhibiting spatial profiles in close agreement with analytical predictions. These results place our system within the broader framework of analogue‐gravity experiments, providing a complementary photonic platform to investigate black‐hole photon‐sphere physics under tabletop conditions and inspiring new approaches to microcavity photonics.

## Graphical abstract

An optical analogue of a Schwarzschild black hole is realized using curved microcavities that preserve light‐like geodesics. A new family of laser modes confined around the photon sphere is identified alongside conventional whispering‐gallery modes. Analytical theory, numerical simulations, and experiments reveal curvature‐induced confinement, enabling photon‐sphere lasing and offering a tabletop platform for black‐hole spectroscopy.

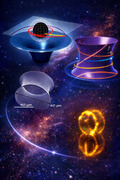

## 1 Introduction

The merger of binary black holes (BHs) releases a tremendous amount of energy into the universe as gravitational waves, first detected in 2015 by the LIGO/VIRGO collaboration [1, 2, 3]. Their spectral signature has provided the opportunity to test general relativity and to indirectly estimate the mass and angular momentum of the BH [4]. The complex frequencies that compose the ringdown spectrum observed at the last stage of BH merger, commonly described by BH perturbation theory [5, 6], would correspond to quasinormal modes intuitively interpreted as damped oscillations of the spacetime, confined around the photon sphere (PS) of the BH [5, 7, 8]. The PS is a spherical region outside the BH, where spacetime is so extremely warped that light trajectories bend and get trapped in a circular orbit around the BH. Orbiting light rays with impact parameters slightly departing from the photon capture radius [9] will either get bent away from or engulfed into the BH. The instability of this classical circular orbit raises a fundamental question: If this region is unstable, why should a gravitational mode be localized or “scarred” around it? This challenge calls for a more precise characterization of these damped modes, including their spatial distribution.

In a controlled laboratory environment, analogue models of gravity provide a powerful route to investigate curved spacetime physics and track down PS dynamics. Following Unruh's seminal proposal of an acoustic black hole in a moving fluid [10], a wide variety of platforms have been developed in which effective metrics emulate horizons and cosmological backgrounds; see Ref. [11] for a review. Experiments in fluids, Bose–Einstein condensates, and nonlinear optical media have reported laboratory analogs of rotational superradiance [12], Hawking radiation [13], the Unruh effect [14], quasinormal ringing [15] and cosmological expansion [16]. Among optical realizations, fiber‐based systems have demonstrated effective event horizons for light pulses, as in the fiber–optical analogue of the event horizon reported in Ref. [17]. For instance, artificial optical materials have been used to achieve light bending [18], wavefront shaping [19] and optical trapping [20], with the index profiles derived from carefully engineered metrics [21].

Complementary to these horizon‐based analogue systems, another table‐top analogue model of gravitational fields consists of a 2D curved surface embedded in 3D Euclidean space [22, 23], which is derived by considering a fixed time coordinate in the four‐dimensional (3+1)D curved spacetime. Based on this purely spatial projection, various optical phenomena have been investigated both theoretically and experimentally [24, 25, 26, 27, 28, 29, 30, 31, 32, 33]. However, removing the time coordinate by setting time as a constant does not preserve the null geodesics in the original (3+1)D black hole spacetime. This calls for a more faithful projection that conserves the Fermat principle of least time.

In this work, we address this question using the Fermat metric, establishing a rigorous analogy between the (3+1)D Schwarzschild spacetime metric and an effective (2+1)D metric on a 2D curved surface that faithfully preserves the original 4D light‐like geodesics. From there, we design the 2D surface of revolution associated with the non‐rotational uncharged Schwarzschild black hole. Because scalar fields in curved spacetime and electromagnetic fields on curved surfaces share the same equation, namely the Klein–Gordon equation, it is possible to mimic the quasinormal modes of ringing BHs on curved optical microcavities within a controllable photonic platform. Here, the term quasinormal modes is used in the standard open‐cavity optics sense to denote resonant modes of a leaky, non‐Hermitian system, while maintaining a close structural analogy with the quasinormal modes strictly defined in general relativity by complex frequencies and outgoing boundary conditions.

In microcavities and microlasers, resonant modes have their backbone along periodic orbits. A famed instance is the whispering‐gallery modes (WGMs), whose ray counterparts undergo polygonal orbits by total internal reflection along the circular boundary. Recently, Song et al. constructed non‐Euclidean microlaser cavities on a Möbius strip and experimentally demonstrated that modes are only sustained on periodic orbits [34]. In these cases, light is confined in the cavity through total internal reflection, with the underlying periodic ray orbit necessarily colliding on the boundary.

Here we show that in non‐Euclidean geometry, the spatial curvature may provide a radically different confining mechanism based on an effective “surface potential” [35], which can possibly trap light without involvement of outer boundaries. High‐Q bottle micro‐resonators based on this concept have been demonstrated on optical glass fiber, with modes localizing on a bulge area [36]. In this example, however, the effective potential is attractive and the corresponding orbits are stable. This contrasts with the a priori repulsive potential induced by curved spacetime of BHs.

We develop an analytic approach to identify the quasinormal modes of BHs optical analogue. We demonstrate the existence of a new family of modes, alongside the well‐known WGMs, which are confined near the photon sphere. Their spectral and spatial characteristics along the photon ring are unveiled analytically and confirmed through finite difference time domain (FDTD) simulations. Schwarzschild laser microcavities are fabricated using 3D direct laser writing in dye‐doped resin. Lasing at the PS is demonstrated. Selective pumping is employed to distinguish between PS modes (PSMs) and WGMs and to map their radial profiles, in excellent agreement with the theoretical prediction. This black hole analogue laser paves the way for designing new types of laser microcavities based on non‐Euclidean surfaces and potential trapping, inspired by celestial objects.

## 2 Theoretical Predictions

### 2.1 The Photon Sphere and Its Stability

The curved spacetime in the vicinity of a spherically symmetric BH can be mathematically depicted by the line element $ds^{2}\equiv g_{\mu \nu }dx^{\mu }dx^{\nu }$ in Schwarzschild coordinates $\left(t,\rho ,\theta ,\varphi \right)$ as

(1) $$\begin{array}{cc} ds^{2} & =-f\left(\rho \right)c^{2}dt^{2}+f^{-1}\left(\rho \right)d\rho^{2}+\rho^{2}d\theta^{2}+\rho^{2}\sin^{2}\theta d\varphi^{2}. \end{array}$$

We consider the Schwarzschild metric, with

(2) $$f\left(\rho \right)=1-\frac{r_{s}}{\rho }.$$

As the simplest spacetime containing a BH, its electric charge, angular momentum and cosmological constant are all vanishing, and its radius $r_{s}$ is determined merely by its mass.

Here, we use the Fermat metric, obtained from the Schwarzschild spacetime in Equation (1) by a conformal transformation, to reduce the problem to an effective (2+1)D geometry while preserving the null geodesics. In the equatorial plane θ=π/2, the associated Fermat metric reads $ds_{F}^{2}=-c^{2}dt^{2}+f^{-2}\left(\rho \right)\;d\rho^{2}+\rho^{2}f^{-1}\left(\rho \right)\;d\varphi^{2}$. Since the time direction is flat and all Christoffel symbols with a time index vanish, the spatial trajectories of null geodesics are governed by its spatial part,

(3) $$ds_{F}^{2}=f^{-2}\left(\rho \right)\;d\rho^{2}+\rho^{2}f^{-1}\left(\rho \right)\;d\varphi^{2}.$$

This two–dimensional Fermat metric is the one we implement optically. It is equivalent to the metric of a surface of revolution r(ρ,φ)=[R(ρ)cosφ,R(ρ)sinφ,H(ρ)] embedded in the 3D Euclidean space, whose first fundamental form has coefficients $E\left(\rho \right)=R^{\prime 2}\left(\rho \right)+H^{\prime 2}\left(\rho \right)$ and $G\left(\rho \right)=R^{2}\left(\rho \right)$. Identifying $E\left(\rho \right)=f^{-2}\left(\rho \right)$ and $G\left(\rho \right)=\rho^{2}f^{-1}\left(\rho \right)$ ensures that Equation (3) coincides with the induced metric on this surface (see Section II, Supporting Information). The Fermat metric ensures that the Fermat principle is also satisfied in the effective (2+1)D Fermat spacetime and that the spatial projections of null geodesics are preserved in the projection process (a detailed derivation can be found in Ref. [41]). In contrast, the traditional approach of assuming constant time [24, 25, 26, 27] fails to reproduce the 4D geodesics.

**FIGURE 1:**
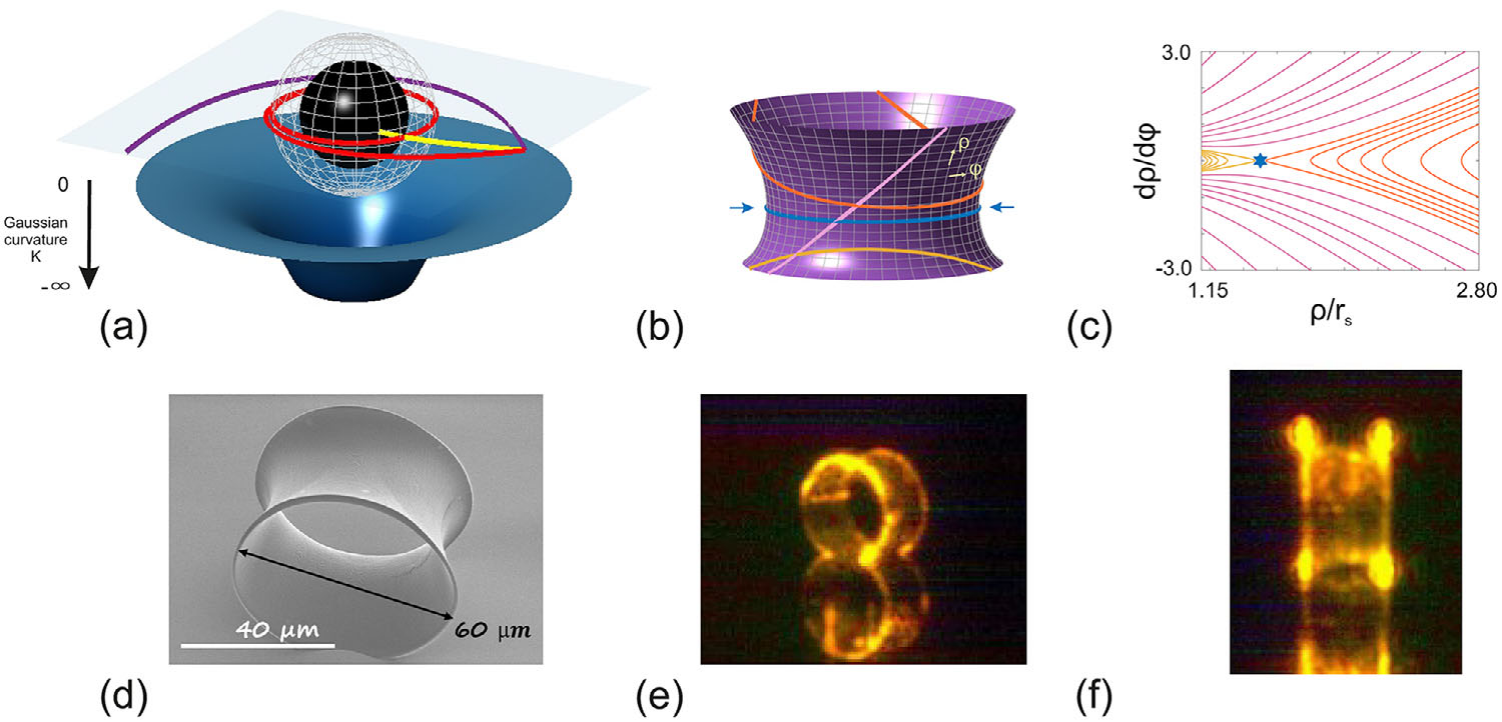
(a) Artist view of a black hole (black sphere) and its Gaussian curvature K (bottom surface), which is always negative and becomes nearly zero at very large distances from the black hole. The spherical mesh materializes the position of its photon sphere. The red line represents a light trajectory trapped on the photon sphere, while trajectories that deviate from it are either absorbed (yellow) or deflected and escape (purple). (b) analogue curved surface of a Schwarzschild black hole with reduced dimensionality, featuring coordinates ρ and φ along the longitudinal and azimuthal directions, respectively. Various trajectories are shown, with the blue solid line (and blue arrows) representing the photon ring orbit. (c) Poincaré surface of section of truncated Schwarzschild surface, where lines correspond to trajectories with same color as in (b). The photon sphere trajectory is marked by the blue star. (d) SEM image of the optical microcavity analogue fabricated by direct laser writing. (e,f) Different optical images of the BH microcavity when uniformly pumped. The microcavity is resting on a glass slide and the recorded image includes both the main object and its reflection.

From there, we construct the corresponding 2D surface of revolution derived from the Schwarzschild metric. Figure 1b illustrates the hyperboloid‐like representation of the Schwarzschild black hole — hereafter referred to as the “Schwarzschild surface.” (For further details on the construction of this curved surface, see Section II, Supporting Information). The lower boundary is conceptually associated with the event horizon at $\rho =r_{s}$ [42]. However, in the surface‐of‐revolution construction the metric coefficient $G\left(\rho \right)=R^{2}\left(\rho \right)=\rho^{2}/f\left(\rho \right)$ diverges as $\rho \to r_{s}$; as a consequence, the height of the curved surface ceases to be real. In this work, we therefore truncate the surface at a finite radius $\rho_{B1}=1.125\;r_{s}$ (see Section II, Supporting Information, for details). An outer truncation at $\rho_{B2}$ is introduced such that the upper boundary is parallel to the lower boundary, preserving rotational symmetry and producing a finite non‐Euclidean cavity [43]. In this work, we fabricate microlasers with this Schwarzschild surface geometry by 3D direct laser writing in a dye‐doped resin, as shown in Figure 1d.

The counterparts of the PS on a curved surface meet the condition obtained from the null or light‐like geodesic equations (see Section I, Supporting Information, for more details)

(4) $$\frac{\rho }{2}\frac{df(\rho )}{d\rho }-f\left(\rho \right)=0.$$

From Equation (4), one finds that the Schwarzschild surface has one unique circular periodic orbit located exactly at its waist (see blue arrows in Figure 1b). On a surface of revolution $r\left(\rho ,\varphi \right)=\left(R(\rho )\cos\varphi ,\;R(\rho )\sin\varphi ,\;H(\rho )\right)$, where R(ρ) denotes the distance to the rotation axis of the embedded surface in Figure 1b, a circular geodesic at fixed ρ satisfies $\Gamma_{\varphi \varphi }^{\rho }=0$, which is equivalent to $R^{\prime }\left(\rho \right)=0$, i.e., the orbit lies at the waist of the surface. Using $f\left(\rho \right)=1-r_{s}/\rho$ in Equation (2), this condition yields the well‐known photon‐sphere radius $\rho_{\mathrm{PS}}=\frac{3}{2}r_{s}$. Thus the Schwarzschild surface has a unique circular periodic orbit located exactly at its waist (see blue arrows in Figure 1b), which corresponds to the photon sphere of the Schwarzschild black hole.

In principle, the stability of closed geodesics on a curved surface is quantified by the Jacobi equation associated with Gaussian curvature, which measures the evolution of the distance between two initially close trajectories,

(5) $$\frac{d^{2}\zeta \left(s\right)}{ds^{2}}+K\left(s\right)\zeta \left(s\right)=0.$$

This means that the geodesic distance ζ(s) between two trajectories oscillates when the Gaussian curvature of the surface K is positive, while it diverges when K is negative. In Section II (Supporting Information), we demonstrate that the Gaussian curvature of a Schwarzschild surface is negative throughout the exterior region $\rho >r_{s}$, which is the region implemented here. This is also illustrated in Figure 1a by the blue surface below the BH. Its PS orbit is therefore unstable. Its instability is also reflected in the Poincaré surface of section [21] shown in Figure 1c. In the Poincaré section, the vertical axis corresponds to the canonical momentum p conjugate to the angular coordinate φ, rather than to sinχ as in conventional billiard plots. Consequently, p is not restricted to the interval [−1,1] and takes values of order unity (see Figure 1c).

### 2.2 The Wave Equation in the Schwarzschild Surface

In the ringdown stage, the evolution of a scalar perturbation Ψ in a black hole background is described by the wave equation (or massless Klein–Gordon equation [44])

(6) $$□\Psi \equiv \frac{1}{\sqrt{g}}\partial_{\mu }\left(\sqrt{g}g^{\mu \nu }\partial_{\nu }\Psi \right)=0,$$

where □ is the d'Alembert operator, $g=\det\left(g_{\mu \nu }\right)$, $g^{\mu \nu }={\left(g^{-1}\right)}_{\mu \nu }$ is the element of the inverse matrix of the original metric. Interestingly, this equation also describes the propagation of electromagnetic waves constrained on a 2D curved surface, i.e., a (2+1)D spacetime, with the curved surface itself embedded in the 3D Euclidean space [22]. The equivalence between these wave equations forms the basis for the analogy between gravitational waves and light waves on curved surfaces. In optics, this can be accomplished by confining light within a thin, curved waveguide, which can be fabricated using direct laser writing technology, as demonstrated later in this article.

### 2.3 Existence of Photon Sphere Modes

Using this analogy, we will now analytically compute the eigenmodes on Schwarzschild surfaces, which serve as the 2D counterparts of modes from actual black holes.

Thanks to the rotational symmetry of the surface of revolution defined in Equation (3), we can write the ansatz

$$\Psi =\rho^{-\frac{1}{2}}{\left(1-\frac{r_{s}}{\rho }\right)}^{-\frac{1}{4}}\psi \left(\rho \right)e^{-il\varphi }e^{ikct}$$

to separate variables (see Section III, Supporting Information, for details). Here, l is the azimuthal quantum number, which has to be an integer to fulfill the periodic condition Ψ(φ)=Ψ(φ+2π), k is the to‐be‐determined eigen wavenumber, and $\psi \left(\rho \right)$ satisfies

(7) $$\frac{d^{2}\psi \left(\rho \right)}{d\rho^{2}}+Q_{s}\left(\rho \right)\psi \left(\rho \right)=0,$$

where

(8) $$Q_{s}\left(\rho \right)=f^{-2}\left(\rho \right)\;\left[k^{2}-V_{\mathrm{eff}}\left(\rho \right)\right],$$

and the effective potential is given by

(9) $$V_{\mathrm{eff}}\left(\rho \right)=\left(l^{2}+\frac{1}{2}\right)\left(\frac{1}{\rho^{2}}-\frac{r_{s}}{\rho^{3}}\right)-\frac{3}{16}{\left(\frac{2}{\rho }-\frac{r_{s}}{\rho^{2}}\right)}^{2}.$$

Equation (7) takes a form reminiscent of the Schrödinger equation.

**FIGURE 2:**
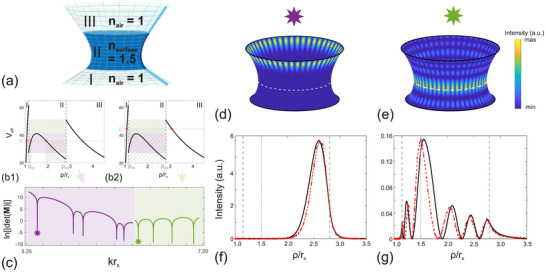
(a) Sketch of the truncated Schwarzschild surface (region II, dark blue) and its tangent truncated cones (regions I and III, light blue) at each boundary. (b) Effective potential of light on Schwarzschild surface for azimuthal number l=25. Figures (b1) and (b2) correspond to two different values of $k^{2}$, indicated by the horizontal black lines. The purple and green area correspond to the $k^{2}$ range of WGMs (b1) and photon sphere modes (b2), respectively. The shadow highlights the oscillatory areas of WKB approximation. In (b1), there exist two turning points, denoted by red dots. (c) Log of absolute determinant of the matrix in Equation (12). Quasinormal modes are identified as sharp dips and are divided into two families: whispering gallery modes (WGM, purple, low $kr_{s}$ values) and photon sphere modes (PSM, green, high $kr_{s}$ values). The mode with the lowest $kr_{s}$ value of each family are highlighted by a star symbol in (c) and plotted in (d–g) figures. (d,f) Mode intensity profile of WGM. (e,g) Mode intensity profile of PSM. Their analytical radial intensity profiles (black solid line) highly conform to simulation results (red dashed line) in (e) and (g).

The eigenmodes of the open Schwarzschild cavity are solutions of Equation (7) in a sandwich structure of air‐surface‐air. For the purpose of modeling, we assume that the radiation emitted from the boundary of the Schwarzschild cavity is distributed along a truncated cone, whose slope is tangent to its generatrix, as depicted by regions I and III in Figure 2a. This assumption is intuitive, as the rotational symmetry of the Schwarzschild surface ensures that radiation follows its longitudes, which form natural paths in flat space. Additionally, the Gaussian curvature of cones is zero, similar to flat space. A general cone can be described by the metric

$$ds_{\text{cone}}^{2}=\left(1+\kappa^{2}\right)d\varrho^{2}+\varrho^{2}d\varphi^{2},$$

where ϱ represents the radius of revolution, and κ denotes the slope. The eigenfrequency spectrum of the entire system can be determined by matching the different regions with appropriate interface conditions. In this work, we focus on transverse magnetic modes, where Ψ represents the component of the electric field perpendicular to the surface at each point. Both Ψ and its first derivative are continuous across the I‐II and II‐III interfaces.

We apply the Wentzel–Kramers–Brillouin (WKB) approximation to solve the Schrödinger‐like Equation (7) [45]. This method yields oscillatory solutions when the spatially varying coefficient $Q_{s}\left(\rho \right)>0$ (represented by the hatched areas in Figure 2b) and produces exponentially decaying or diverging solutions when $Q_{s}\left(\rho \right)<0$. The points where $Q_{s}\left(\rho \right)=0$ are known as turning points, where the WKB approximation breaks down. In such cases, the modified Airy function method can be employed, as it effectively handles the behavior at turning points [46]. The sign of $Q_{s}\left(\rho \right)$ at any given position ρ is determined by the variable k. As illustrated in Figure 2b, the effective potential $V_{\text{eff}}$ on the Schwarzschild surface resembles a hill, with its peak precisely located at the PS, drawing an analogy with the potential in quantum mechanical systems. Conversely, in both tangent cones, $V_{\text{eff}}$ decreases as the distance from the I‐II and II‐III interfaces increases. It is important to note that our approach follows the “modes‐of‐the‐universe” framework [47], where the field is quantized within the Schwarzschild cavity and its tangent truncated cones. Since energy is conserved throughout the system, the corresponding wavenumbers k are real. This treatment captures the spatial structure and resonance positions of the open system, whereas the finite lifetimes of the modes arise from radiation loss and are quantified numerically and experimentally.

To begin, we search for eigenmodes where $k^{2}>V_{\text{eff}}^{\left(II\right)}\left(\rho_{\text{PS}}\right)$, which corresponds to the case of Figure 2 (b2). In this case, we also restrict $k^{2}$ such that $k^{2}<\min\left(V_{\text{eff}}^{\left(I\right)}\left(\rho_{B1}\right),V_{\text{eff}}^{\left(III\right)}\left(\rho_{B2}\right)\right)$, ensuring the presence of turning points in both Region I and Region III. Here, $\rho_{\mathrm{PS}}$ denotes the photon‐sphere radius, i.e., the position of the maximum of $V_{\mathrm{eff}}^{\left(\mathrm{II}\right)}$ in the central Schwarzschild region, while $\rho_{B1}$ and $\rho_{B2}$ are the radial coordinates of the inner and outer truncation boundaries where the Schwarzschild surface is attached to its tangent cones (boundaries of Region I–II and Region II–III respectively in Figure 2a). These turning points divide each region into a decaying area (toward the interface) and an oscillatory area (extending toward infinity). As a consequence, QNMs are more confined between the interfaces, and thus more promising for experimental demonstration. The wave function in the cavity (region II) can be written as

(10) $$\psi^{\left(II\right)}\left(\rho \right)=C_{2+}\phi_{2+}\left(\rho \right)+C_{2-}\phi_{2-}\left(\rho \right),$$

where

(11) $$\phi_{2\pm }\left(\rho \right)={\left[Q_{s}^{\left(II\right)}\left(\rho \right)\right]}^{-\frac{1}{4}}\exp\left(\pm i\int_{\rho_{B1}}^{\rho }\sqrt{Q_{s}^{\left(II\right)}\left(\rho^{\prime }\right)}d\rho^{\prime }\right),$$

and $C_{2+}$ and $C_{2-}$ are to‐be‐determined constant coefficients. The stitch of the wave function in these three regions, along with to‐be‐determined coefficients, can be written in matrix form as (see the Method section)

(12) $$M\left(\begin{array}{c} C_{1}\\ C_{2+}\\ C_{2-}\\ C_{3} \end{array}\right)=0,$$

with $C_{1}$ and $C_{3}$ being the coefficients in Region I and III of the evanescent tail outside the cavity. To obtain nontrivial solutions, the determinant of the matrix must vanish, i.e., $\det\left(M\right)=0$. As k is the only variable in matrix M, eigen‐wave numbers are the zeros of $\det\left(M\right)$, represented as sharp dips in Figure 2c. Given a representative l=25, four solutions are found in the green area of Figure 2c, associated with modes confined by the effective potential within the Schwarzschild cavity. In what follows, the theoretical radial intensity profiles plotted in Figure 2f,g are defined as $I\left(\rho \right)\propto {\left|\Psi \left(\rho \right)\right|}^{2}=\rho^{-1}f{\left(\rho \right)}^{-1/2}{\left|\psi \left(\rho \right)\right|}^{2}$, where Ψ is the physical field on the Schwarzschild surface and f(ρ) is given by Equation (2). For the sake of comparison with simulations, these profiles are normalized to their maximum value. The first mode is shown in Figure 2e,g, with maximum intensity near the PS. We identify this mode as the fundamental PS mode. Indeed, the envelope of PS modes in Equation (11) conforms to ${\left[Q_{s}^{\left(II\right)}\left(\rho \right)\right]}^{-\frac{1}{4}}$, peaking at the PS, while the actual peak of intensity might deviate from the PS due to the oscillatory term. Higher orders of PS modes, with faster oscillations, are reported in Figure S2 (Supporting Information).

For quasinormal modes with frequencies down to $k\in \left(\max\left(V_{\text{eff}}^{\left(II\right)}\left(\rho_{B1}\right),V_{\text{eff}}^{\left(II\right)}\left(\rho_{B2}\right)\right),V_{\text{eff}}^{\left(II\right)}\left(\rho_{\text{PS}}\right)\right)$, corresponding to the case of Figure 2 (b1), two turning points appear in the Schwarzschild cavity, dividing region II into three areas. PS modes can no longer be sustained as the PS falls within the decaying area. Instead, oscillating areas are situated near the two boundaries, yielding the corresponding quasinormal modes akin to WGMs. A typical WGM solution of Equation (10) is shown in Figure 2d,f for comparison. We show in Figure S3 (Supporting Information) the wave functions of all five WGM solutions found in the purple region of Figure 2c.

### 2.4 Numerical Simulations of Quasi‐Normal Modes

To test our theoretical prediction, we perform finite difference time domain (FDTD) simulations and numerically exhibit the eigenmodes in the Schwarzschild cavity. As full‐wave simulations on the curved surface embedded in 3D Euclidean space can be computationally expensive, here we implement the simulations on its conformally transformed gradient‐index plane [50]. Thus the Schwarzschild cavity is represented by a disk of radius 5μm in Cartesian coordinates (x,y), with a radially varying discretized index of refraction. Perfectly matched layers (PML) surround the simulated area, acting as the open boundary at the interface II‐III. A TM‐polarized pulse with central frequency 210 THz (corresponding to wavelength 1.4 μm) and pulse length 30 fs is applied, and the temporal evolution of the electric field is recorded at different positions. The 2D simulations are performed by the Ansys Lumerical FDTD software.

**FIGURE 3:**
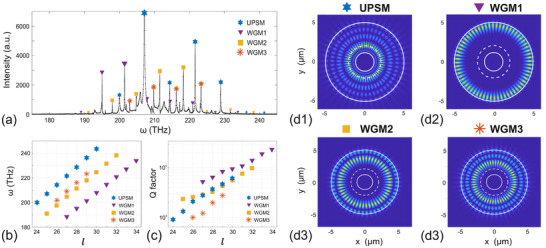
(a) Numerical spectrum. Four families of quasinormal modes, including the fundamental unstable photon sphere mode (UPSM) and three WGMs, are identified from (a). (b) Frequencies and (c) Q factors of all the identified modes in (a) with different angular numbers. (d1–d4) illustrate the typical intensity distribution of these modes. The color maps show $|E_{z}{\left(x,y\right)|}^{2}$ in the conformally transformed 2D plane, where (x,y) are Cartesian coordinates in units of μm. The PS orbit, mapped to this plane, is indicated by a dashed white circle. Each panel corresponds to the same mode highlighted in (b,c), so that the corresponding azimuthal number l is given by the abscissa of the marked point in those plots.

By Fourier transforming the sum of all collected time signals, we obtain the power spectrum in Figure 3a. Spectral peaks correspond to resonances of the structure, of which the intensity distribution can be computed. Intensity distribution of four typical quasinormal modes are shown in Figure 3b. Different families of modes are identified from their intensity distribution, corresponding to PS modes when peaked near the waist [Figure 3(b1)], and WGMs when confined near the system boundaries, [Figure 3(b2)]. The radial intensity distribution can be extracted and compared with theoretical calculations, as illustrated in Figure 2f,g. The radial intensity distribution can be extracted and compared with the theoretical profiles illustrated in Figure 2f,g. In the FDTD simulations, the intensity is computed as $|E_{z}{\left(x,y\right)|}^{2}$ on the conformally transformed 2D plane. For each mode, we then extract a radial line profile along the azimuthal direction where the intensity on the orbit attains its maximum. Because the modes are periodic in φ, this cut is representative, and the resulting radial dependence is denoted I(ρ), directly comparable to the theoretical intensity ${\left|\Psi \left(\rho \right)\right|}^{2}$. The simulated intensity profiles, represented by red dashed lines, show a high degree of consistency with the analytical results, providing strong validation for the theoretical predictions.

The resonance frequencies of all modes [48] in Figure 3a are plotted in Figure 3b as a function of their azimuthal number, l, found from the intensity spatial distribution of each of these modes. For the relatively large azimuthal numbers considered here (24≤l≤34), different families of modes, including higher‐order WGMs, exhibit an approximately linear dependence on l, as expected from the azimuthal quantization condition $l\approx n_{\mathrm{eff}}kR_{\mathrm{eff}}$ ($n_{\mathrm{eff}}$ is the effective refractive index and $R_{\mathrm{eff}}$ the effective radius experienced by the mode.)

Finally, we are able to extract the quality factor, $Q=\omega_{0}/\Delta \omega$, of each mode by fitting each spectral peak in Figure 3a with a Lorentzian function [51, 52], which provides direct access to the imaginary part of the complex resonance frequency associated with radiative losses. The Q factors for different families of modes are shown in Figure 3c as a function of their azimuthal number, l. The Q factors of the PS mode family are lower than those of the first‐order WGM. This difference arises because WGMs are strongly confined near the outer boundary by total internal reflection, whereas the potential‐induced confinement of PSMs does not fully suppress their radiation loss from the truncated structure, as shown in Figure 3(b1). Nevertheless, the Q factors of PS modes are comparable to or even exceed those of higher‐order WGMs (Figure 3c), highlighting the effectiveness of curvature‐induced light confinement in the propagation layer.

## 3 Experimental Demonstration

We now proceed to experimentally validate our theoretical predictions on the existence of modes at the PS. The Schwarzschild surface is fabricated using advanced 3D direct laser writing, utilizing Nanoscribe's two‐photon polymerization technology. Given the relatively short‐lived nature of the predicted optical quasinormal modes, we propose investigating the corresponding lasing modes in a dye‐doped organic structure. This approach would help mitigate the losses and leverage the gain to selectively amplify the modes of the passive system. A SEM image of the fabricated curved‐surface microcavity laser is shown in Figure 1d. The fabrication process, the sample details and the experimental setup are described in the Method section.

### 3.1 Experimental Evidence of Photon Sphere Modes

**FIGURE 4:**
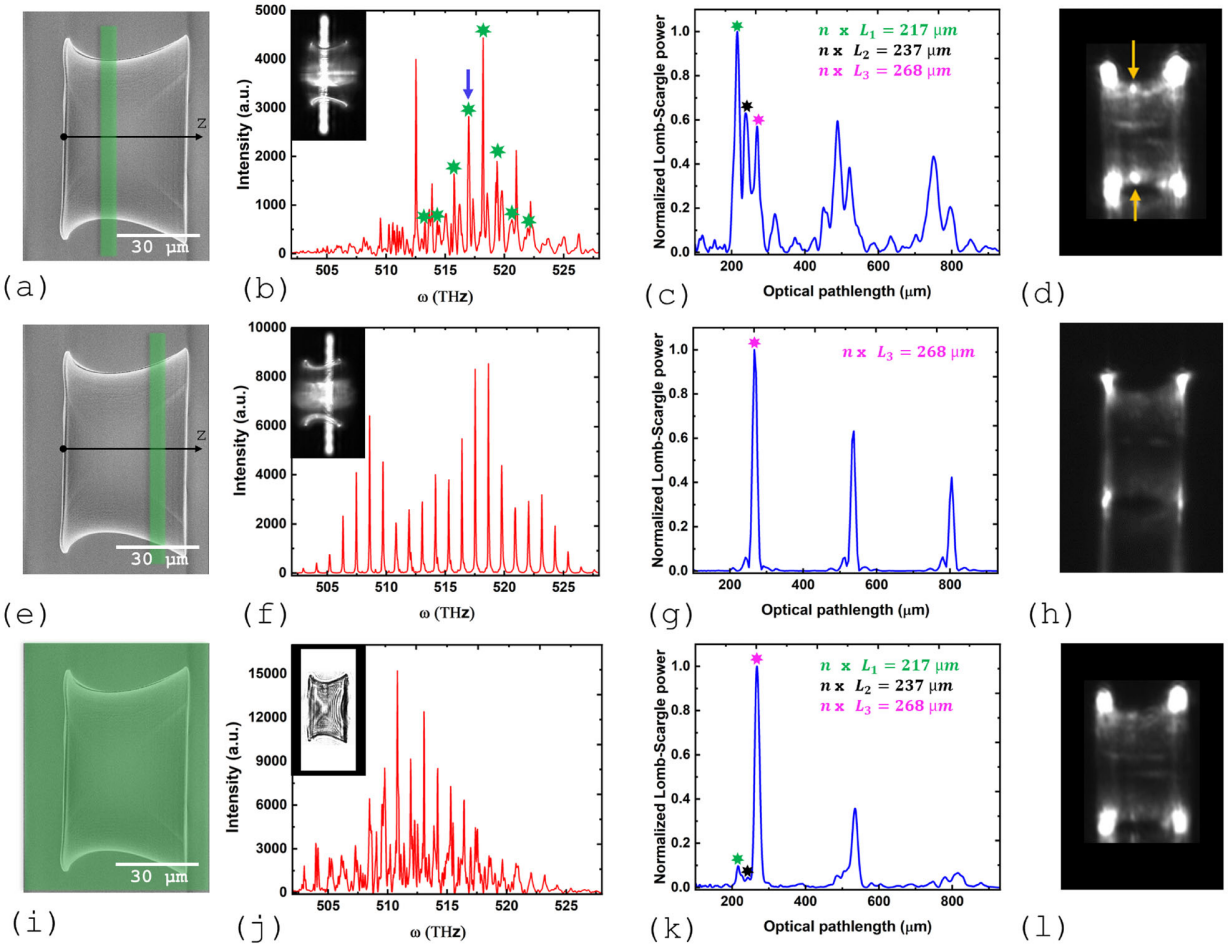
(a) SEM images of the Schwarzschild black hole microcavity laser resting on a glass plate. The z‐axis represents the axis of the surface of revolution. The 4 μm‐wide and 95 μm‐long pump stripe, illustrated by the green rectangle, is placed at the waist to selectively excite the photon sphere mode. (b) Emission spectrum of the microstructure when locally pumped near the waist. PS modes are indicated by stars. Inset: top view of the structure with the pump stripe at the waist. (c) Lomb–Scargle periodogram of the emission spectrum in (b). Three peaks are identified in the first group at $nL_{1}$ = 217 μm, $nL_{2}$ = 237 μm, and $nL_{3}$ = 268 μm. (d) Direct observation of the black hole analogue laser emission (the pump wavelength is blocked by a notch filter). Yellow arrows point to the PS lasing mode at the waist. (e) Selective excitation of the WGM confined near the sample edge. (f) Emission spectrum of the laser microcavity when selectively pumped near the WGM location close to the sample edge. Inset: the pump stripe's position. (g) Lomb‐Scargle periodogram of the emission spectrum in (f). Only the peak at $nL_{3}$ = 268 remains. (h) Direct observation of the WGM laser emission. Emission at the waist has disappeared. (i) Uniform pumping of the microcavity laser with a 50 μm‐wide and 95 μm‐long rectangular pump profile. (j) Emission spectrum of the microstructure when uniformly pumped. The inset shows the top view of the structure. (k) Lomb–Scargle periodogram of the emission spectrum in (j), identifying a dominant peak at $nL_{3}=268\mu m$ along with a feeble signature of the PS modes family and the WGMs confined near the left sample edge. (l) Direct observation of lasing microcavity under uniform pumping.

With their high Q‐factor, WGMs are anticipated to dominate the emission spectrum, as they have a lower lasing threshold than PS modes. To enhance the likelihood of observing PS lasing modes, we propose to pump the microcavity locally, near the PS, away from the edges where WGMs are predominantly confined. Selective pumping has been proposed earlier to selectively excite lasing modes and achieve single mode lasing in random lasers [53]. The pump intensity profile of a frequency‐doubled Q‐switched Nd:YAG laser at 532 nm is shaped by reflection on a spatial light modulator into a 4 μm‐wide and 95 μm‐long narrow stripe positioned at the waist, as shown in Figure 4a. The top view of the structure with the pump stripe aligned along the waist is shown in the inset of Figure 4b. At sufficiently high pump energy, multimode laser emission is observed as shown in Figure 4b. Following the Lomb–Scargle method, we identify regular spacings between spectral lines in the measured emission spectrum by fitting sinusoidal modulations as a function of frequency, thereby revealing the optical path length associated with families of cavity modes sharing the same azimuthal number, as shown in Figure 4c. The group refractive index, n=1.56, is obtained from an independent measurement using a reference cuboid microlaser (see Figure S4, Supporting Information). Three distinct peaks are seen in the first group, indicating the presence of lasing modes associated with three distinct orbits. The dominant peak corresponds to an optical path length $nL_{1}$ = 217 μm. Assuming the orbits are circular, we find a corresponding diameter $D_{1}$ = 44.3 μm, which closely matches the waist diameter, $D_{W}$ = 44.5 ±1 μm, providing the first experimental evidence that a family of modes exists at the photon ring of the optical analogue of Schwarzschild black hole. The corresponding lasing peaks are identified in the emission spectrum of Figure 4b and are indicated by stars in the figure. They form a comb of equally‐spaced lasing frequencies associated with modes with increasing azimuthal number, l, as predicted by the theory (Figure 3c). PS lasing is directly observed under the microscope, as shown in Figure 4d, where two lasing spots are seen precisely at the location of the PS.

The two additional peaks seen in the Lomb–Scargle periodogram at $nL_{2}=237\;\mu m$ and $nL_{3}=268\;\mu m$ are identified as families of modes on circular orbits with $D_{2}=48.4\;\mu m$ and $D_{3}=54.7\;\mu m$. These modes are confined near the sample edges, at $D_{L}$ = 48.9 ±1 μm and $D_{R}$ = 60.0 ±1 μm, and are believed to be WGMs. This is confirmed by moving the pump strip to the positions of these modes. For instance, Figure 4f,g respectively shows the emission spectrum and the periodogram when pumping near the position of orbit $L_{3}$. The position of the pump stripe on the structure is shown in Figure 4e and in the inset of Figure 4f. Only the third peak at $nL_{3}=268\;\mu m$ remains in the periodogram, reflecting the spectral comb of Figure 4f. As anticipated, the lasing spots at the PS have vanished as can be seen in Figure 4h. Also note that, irrespective of selective pumping the orbital position L3, both edges show considerable light emission. This is because, the excited WGM corresponds to a global orbit circulating around the structure. Once lasing is established, the modal intensity appears at both edges, even when only one edge is pumped. When the pump is moved to other positions, additional WGMs are observed near the sample edges (not shown), corresponding to higher‐order WGMs, as predicted by theory (see Figure S2, Supporting Information).

In contrast to selective excitation, uniform pumping of the microcavity laser would be dominated by WGMs. The emission spectrum with a 50 μm‐wide and 95 μm‐long rectangular pump profile (Figure 4i) is presented in Figure 4j. The corresponding Lomb–Scargle periodogram (Figure 4k) reveals a dominant peak at 268 μm corresponding to the previously identified WGM. The dominance of this WGM is attributed to its lowest loss. So this mode reaches threshold first and dominates the spectrum. A feeble signature of the PS mode and the WGM confined near the sample edge at $D_{L}$ = 48.9 ±1 is also present. An image of the lasing microcavity under uniform pumping is shown in Figure 4l (see also Figure 1e,f).

### 3.2 Photon Sphere Mode Profile Measurement

**FIGURE 5:**
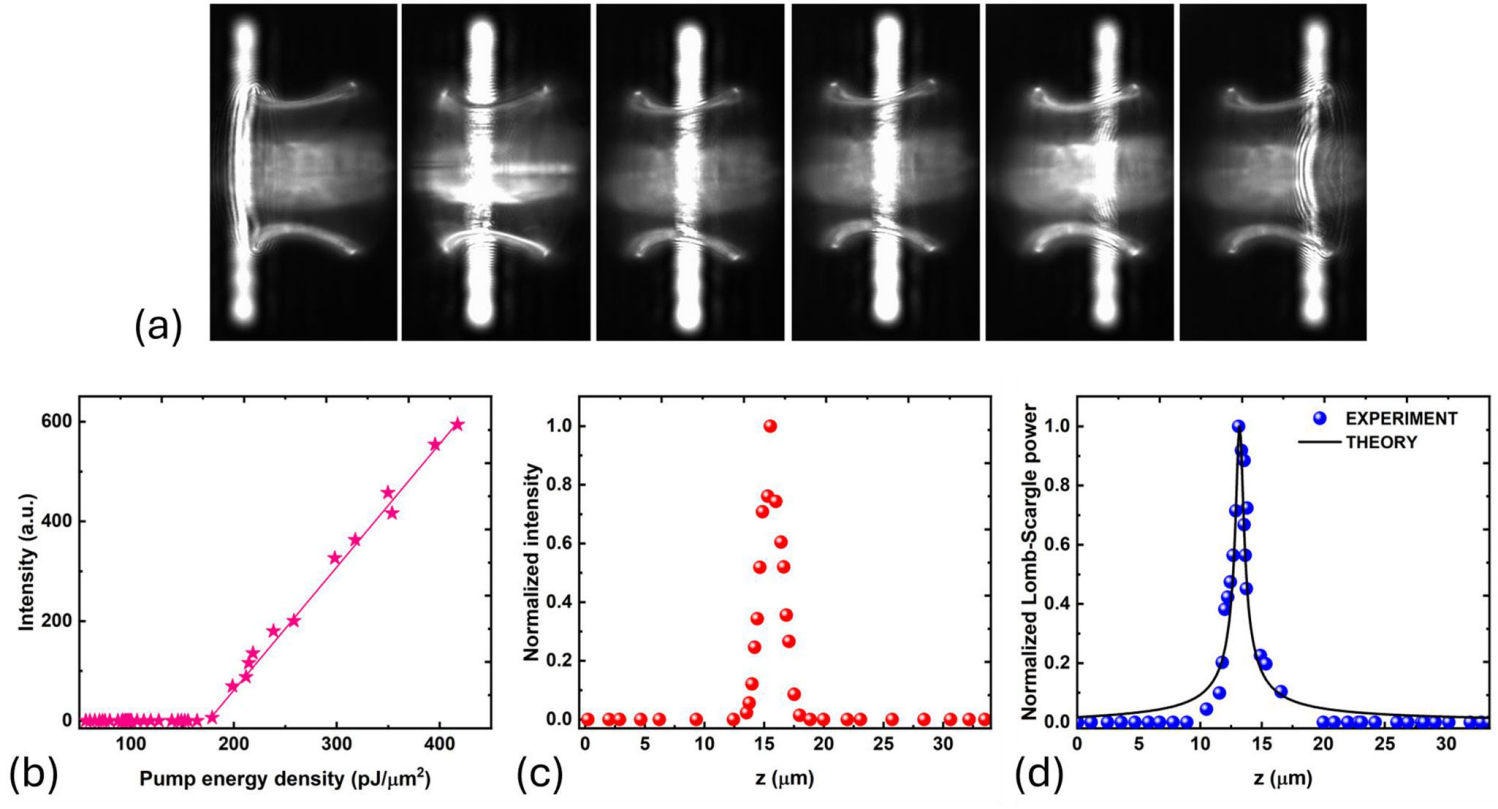
(a) Scanning of the pump stripe along the axis of the surface of revolution (z‐axis). Few instances of the scanning process. (b) Laser characteristic of the PS lasing mode at 580.3 nm under selective pumping. (c) Spatial intensity distribution of the PS mode, derived from the emission spectrum at 580.3 nm (indicated by the arrow in Figure 4b), as the pump stripe is scanned along the z‐axis. (d) Amplitude of the peak at $nL_{1}$ = 217 μm (corresponding to PS modes) in the Lomb–Scargle periodogram of Figure 4c as the pump stripe is scanned along the z‐axis (blue symbols). The experimental data are compared to the theoretical prediction of the spatial intensity profile of the PS modes with azimuthal number l=377 (black line).

We now focus on a specific PS lasing mode that peaks at 580.3 nm and is indicated by the arrow in Figure 4b. The peak intensity as a function of pump energy is shown in Figure 5b, indicating a laser threshold of 180 pJ/μ $m^{2}$ for this particular mode. To characterize the spatial distribution of this PS mode along the sample axis (z‐axis), we record the peak intensity at 580.3 nm as we slide the pump stripe across the sample in steps of 220 nm. Few instances of the scanning process are shown in Figure 5a. After applying Lucy–Richardson deconvolution, we obtain the spatial distribution of this particular mode shown in Figure 5c. The strong confinement observed around the waist position confirms unequivocally that the trapping mechanism is driven by the curvature‐induced potential rather than by reflection on the sample edges. The same scanning process is repeated, but this time, the peak amplitude at $nL_{1}$ = 217 μm in the periodogram of Figure 4c corresponding to the family of PS modes is plotted against the pump stripe position (Figure 5d). This effectively performs a weighted spatial averaging over the intensity profile of all PS modes. Remarkably, this measurement aligns closely with our theoretical predictions when PS modes are calculated for the actual sample dimensions and corresponding azimuthal number. The azimuthal number l in the experiment can be estimated using the relation $\pi nD_{W}=l\lambda$, where $D_{W}$ is the waist diameter. We find that l ranges from 371 to 383 for all lasing modes identified in (Figure 4b). Considering the average value, l=377, we then calculate analytically the corresponding fundamental PS mode and compare its spatial profile to the measured one (black line in Figure 5d): Excellent agreement in position and spatial extension is obtained between experiment and theory.

## 4 Conclusion

To summarize, we have proposed a dimensionality‐reduced optical analogue of a 4D Schwarzschild black hole, which genuinely preserves its light‐like geodesics and shares with it the same governing equations. Our analytical investigation, supported by numerical simulations, predicts the existence of modes at the PS of the optical Schwarzschild surface, and we compute their spectral and spatial characteristics. These QNMs, responsible for gravitational radiation in the universe, are visualized here for the first time in 3D‐printed Schwarzschild laser microcavities. Selective pumping allows us to discriminate between curvature‐induced potential‐confined PS modes and cavity‐trapped WGMs. These modes are confined due to spatial curvature, rather than cavity boundaries, offering new insights into mode confinement mechanisms.

Since the metric in Equation (1) can take different forms, such as by introducing a non‐zero cosmological constant, the quantity and position of PSs can be engineered. This paves the way for designing optical cavities and laser devices with novel functionalities.

We believe that black hole analogs, like the one we introduced, can shed new light on current questions in gravitational physics. In particular, perturbations near the event horizon offer a way to test Einstein's theory of general relativity. Here, we present the spatial distribution of the wavefunction, which can be crucial for investigating mode stability [54, 55]. The stability of the obtained quasinormal mode spectrum can be further examined using methods such as the pseudospectrum approach [54], which is relevant to gravitational‐wave analysis [56] and could help in detecting fundamental modes and overtones [57, 58]. The analogue model discussed in this work may facilitate the study of a wide range of celestial events and phenomena, including black hole spectroscopy [59], Lyapunov exponents [60], wave dynamics, Hawking radiation, superradiance, and black hole evaporation [61]. These analogue systems also provide physical testbeds for exploring ideas like gravitational wave echoes [62], whose time‐delayed signatures can be designed and observed by constructing the trapping cavity formed between a photon sphere and a reflective inner surface. In the future, such an approach could lead to studies of backreaction and nonlinear QNM interactions [63, 64, 65, 66, 67].

## 5 Methods

### 5.1 Derivation of Coefficient Matrix **M**

Elements of matrix M are wave functions of each region at the boundaries. In Region I and III, as the turning points are far enough from the boundaries, the field decays to a negligible amplitude before reaching the oscillatory region. Therefore we write the wave functions in the decaying areas as

(13) $$\phi^{\left(I\right)}\left(\rho \right)=C_{1}\phi_{1-}\left(\rho \right),\phi^{\left(III\right)}\left(\rho \right)=C_{3}\phi_{3-}\left(\rho \right),$$

where $C_{1}$ and $C_{3}$ are to‐be‐determined coefficients,

(14) $$\phi_{1-}\left(\rho \right)={\left[Q_{s}^{\left(I\right)}\left(\rho \right)\right]}^{-\frac{1}{4}}\exp\left(\int_{\rho_{B1}}^{\rho }\sqrt{-Q_{s}^{\left(I\right)}\left(\rho^{\prime }\right)}d\rho^{\prime }\right),$$

(15) $$\phi_{3-}\left(\rho \right)={\left[Q_{s}^{\left(III\right)}\left(\rho \right)\right]}^{-\frac{1}{4}}\exp\left(-\int_{\rho_{B2}}^{\rho }\sqrt{-Q_{s}^{\left(III\right)}\left(\rho^{\prime }\right)}d\rho^{\prime }\right).$$

Stitching of wave functions Equations (10), (11), (13)‐(15) at the boundaries leads to

(16) $$\begin{array}{c} M=\left(\begin{array}{cccc} \phi_{1-}\left(\rho_{B1}\right) & -\phi_{2+}\left(\rho_{B1}\right) & -\phi_{2-}\left(\rho_{B1}\right) & 0\\ \frac{d\phi_{1-}}{d\rho }\left(\rho_{B1}\right) & -\frac{d\phi_{2+}}{d\rho }\left(\rho_{B1}\right) & -\frac{d\phi_{2-}}{d\rho }\left(\rho_{B1}\right) & 0\\ 0 & \phi_{2+}\left(\rho_{B2}\right) & \phi_{2-}\left(\rho_{B2}\right) & -\phi_{3-}\left(\rho_{B2}\right)\\ 0 & \frac{d\phi_{2+}}{d\rho }\left(\rho_{B2}\right) & \frac{d\phi_{2-}}{d\rho }\left(\rho_{B2}\right) & -\frac{d\phi_{3-}}{d\rho }\left(\rho_{B2}\right) \end{array}\right). \end{array}$$

### 5.2 Sample Fabrication

The laser microcavity is 3D‐printed in dye‐doped resin, using two‐photon polymerization with commercial Nanoscribe lithography GT system. IP‐G is used as the Nanoscribe resist and is doped with Pyrromethene 597 (0.5 % wt) to incorporate gain. Laser printing begins within the glass substrate to securely anchor the microcavity. A dozen of BH microlasers are fabricated on the same glass slide with very good reproducibility. SEM image of a Schwarzschild BH microlaser is shown in Figure 1d, Figure 4a,e,i and Figure 5a. The waist of the structure has a diameter $D_{W}$= 44.5 ±1 μm. The diameters of the left (relative to the z‐axis defined in Figure 4a) and right circular edges are $D_{L}$ = 49 ±1 μm and $D_{R}$ = 60 ±1 μm, respectively.

### 5.3 Experimental Setup

The experimental apparatus includes a reflective spatial light phase modulator (SLM) (HES 6001 from Holoeye, pixel size 8.0 μm), which serves as a secondary display for the computer and can receive a grayscale image of the desired shape as needed. To selectively pump specific areas of the laser microcavity, a MATLAB‐generated rectangular grayscale image with a uniform value of 255 is sent to the SLM. The pump laser (Ekspla PL2230 with λ = 532 nm, maximum output energy of 28 mJ, pulse duration of 20 ps, repetition rate of 10 Hz) reflects this grayscale image from the SLM. Furthermore, the SLM rotates the pump laser's polarization by 90 degrees in regions where the grayscale value is non‐zero. A polarizer positioned after the SLM is optimized to achieve the corresponding amplitude modulation. The pump polarization is aligned with the cavity axis of rotation (z‐axis). The setup also includes two sCMOS cameras, one attached to a zoom lens, the other to a fixed‐stage microscope (Zeiss AxioExaminer A1) for imaging the curved‐surface cavity from both the side and the top. The shaped pump beam is directed upward, allowing top imaging under the microscope to ensure precise alignment of the pump strip perpendicular to the laser microcavity's axis and to monitor mode profile as it is moved along the structure. Lasing emission is collected by a microscope objective (20 × from Thorlabs) connected to a high‐resolution imaging spectrometer (iHR550 from Horiba, 2400 ${\mathrm{mm}}^{-1}$ density grating, spectral resolution of 20 pm and Synapse camera) via a multimode fiber.

### 5.4 Data Analysis

The Lomb–Scargle routine of Matlab, plomb.m, has been used, which yields a Fourier‐like power spectrum, but with a better accuracy in the peak positions and a better signal‐to‐noise ratio than usual discrete Fourier transform algorithms.

The Lucy–Richardson deconvolution is a widely used algorithm for image deblurring. The routine deconvlucy.m from Matlab has been used here to improve the resolution of the mode profile, beyond the width of the pump stripe.

## Author Contributions

P.S. and M.L. conceived the idea, designed the research and supervised the project. C.X. performed all numerical simulations, analytical calculations and theoretical analysis. A.S., M.L. and N.B.K. developed the experimental setup, conducted the experiments and performed data analysis. C.X. and A.S. drafted the initial manuscript. C.L. and D.D. fabricated the structures. L.Z., L.W., O.B. actively participated in the discussions and provided valuable insights. All authors reviewed and approved the final manuscript.

## Conflicts of Interest

The authors declare no conflicts of interest.

## Supporting information


**Supporting File**: advs74652‐sup‐0001‐SuppMat.pdf.

## Data Availability

The data that support the findings of this study are available from the corresponding author upon reasonable request.
