# Supplementary material for: Photon‐Sphere Modes in Curved Optical Microcavities: A Black‐Hole Analogue Laser
Source: Adv Sci (Weinh). 2026 Mar 6;13(28):e17466. doi: 10.1002/advs.202517466 (PMC13185855; doi:10.1002/advs.202517466)
Supplement: Supplementary file 1 — Supporting File: advs74652‐sup‐0001‐SuppMat.pdf. [file ADVS-13-e17466-s001.pdf]

Supplementary Information: Photon-Sphere Modes in Curved Optical Microcavities: a Black-Hole Analogue Laser

Chenni Xu[1§], Aswathy Sundaresan[1§], Nazire-Begüm Kazkal[2], Clement Lafargue[3], Lior Zarfaty[1], Li-Gang Wang[4], Ofek Birnholtz[1], Dominique Decanini[2], Melanie Lebental[2], and Patrick Sebbah[1*]

[1] *Department of Physics, The Jack and Pearl Resnick Institute for Advanced Technology, Bar-Ilan University, Ramat-Gan 5290002, Israel*

[2] *Université Paris-Saclay, CNRS, Centre de Nanosciences et de Nanotechnologies, 91120, Palaiseau, France*

[3] *Laboratoire Lumière, Matière et Interfaces (LuMIn) CNRS, ENS Paris-Saclay, Université Paris-Saclay, CentraleSupélec, 91190 Gif-sur-Yvette, France and*

[4] *School of Physics, Zhejiang University, Hangzhou 310058, China*

I. GEODESICS ON SCHWARZSCHILD SURFACES

On an arbitrary two-dimensional curved surface with a given metric g_{ij}, geodesics follow

$$\frac{d^2x^i}{d\xi^2} + \Gamma^i_{jk}\frac{dx^j}{d\xi}\frac{dx^k}{d\xi} = 0. \tag{S.1}$$

Here ξ is the affine parameter which could be taken as the line element s, $\Gamma^i_{jk} = \frac{1}{2}g^{i\ell}g_{\ell j},k + g_{\ell k},j - g_{jk},\ell)$ is the Christoffel connection with $g^{i\ell}$ being elements of the inverse of the metric tensor $\mathbf{g}$ and "," denoting first-order derivatives, $i, j, k, \ell = 1, 2$, and Einstein's summation convention is used. By plugging the metric of Eq. (3) into Eq. (S.1) and performing basic algebra, we obtain equations below,

$$\frac{d^2\rho}{ds^2} - \frac{1}{f(\rho)}\frac{df(\rho)}{d\rho}\left(\frac{d\rho}{ds}\right)^2 + \left[\frac{\rho^2}{2}\frac{df(\rho)}{d\rho} - \rho f(\rho)\right]\left(\frac{d\varphi}{ds}\right)^2 = 0, \tag{S.2}$$

$$\frac{d^2\varphi}{ds^2} + 2\left[\frac{1}{\rho} - \frac{1}{2f(\rho)}\frac{df(\rho)}{d\rho}\right]\frac{d\rho}{ds}\frac{d\varphi}{ds} = 0. \tag{S.3}$$

Thanks to its rotational symmetry, geodesics on surfaces of revolution conserve angular momenta. This conserved quantity, ε, can be obtained by solving Eq. (S.3) as

$$\rho^2 f^{-1}(\rho)\,\frac{d\phi}{ds} = \varepsilon. \tag{S.4}$$

For any free trajectory (i.e., without external force, collision, etc.), the quantity ε remains constant once the initial position and direction are given. Using Eq. (S.4) together with the normalization of the tangent vector for a geodesic parametrized by the arc-length s of the two-dimensional Fermat metric,

$$g_{ij}\,\frac{dx^i}{ds}\frac{dx^j}{ds} = 1,$$

and inserting the Schwarzschild surface metric $ds^2 = f^{-2}(\rho)\,d\rho^2 + \rho^2 f^{-1}(\rho)\,d\phi^2$, we obtain

$$\left(\frac{d\rho}{ds}\right)^2 = f^2(\rho)\left[1 - \frac{\varepsilon^2}{\rho^2 f(\rho)}\right]. \tag{S.4}$$

Therefore the radial evolution can be written as

$$\frac{d\rho}{ds} = \eta\, f(\rho)\sqrt{1 - \frac{\varepsilon^2}{\rho^2 f(\rho)}}, \tag{S.5}$$

*patrick.sebbah@biu.ac.il

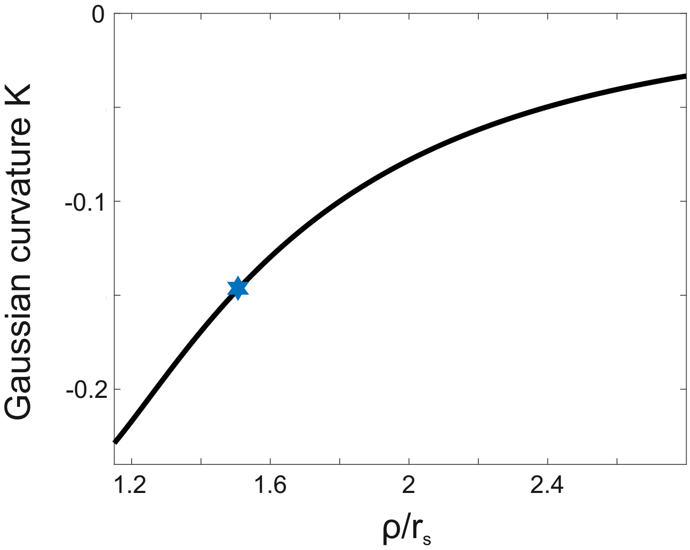

FIG. S1: Gaussian curvature K of a Schwarzschild surface. The blue hexagon denotes the unstable photon sphere.

where $\eta = 1$ corresponds to the case with positive initial longitudinal speed, i.e. $\frac{d\rho}{ds}\Big|_{\text{initial}} > 0$, and $\eta = -1$ corresponds to $\frac{d\rho}{ds}\Big|_{\text{initial}} < 0$. Note that $\eta = 1$ and $\eta = -1$ refer to two different geodesics. With Eqs. (S.4) and (S.5), one readily has

$$d\phi = \frac{\varepsilon}{\eta\,\rho^2}\,\frac{d\rho}{\sqrt{1-\varepsilon^2/[\rho^2 f(\rho)]}}. \tag{S.6}$$

by which trajectories of geodesics are determined.

II. GAUSSIAN CURVATURE OF SURFACES OF REVOLUTION

Points on an arbitrary surface of revolution can be written as $\mathbf{s} = [R(\rho)\cos\varphi, R(\rho)\sin\varphi, H(\rho)]$. Geometrically, at each point, Gaussian curvature is viewed as product of the two principal curvatures $\varkappa_1$ and $\varkappa_2$, where the two principal curvatures are the reciprocal of principal radii of curvature, which are the radii of maximal and minimal tangent circles. Mathematically, Gaussian curvature can be obtained by

$$K = \frac{eg - f^2}{EG - F^2}, \tag{S.5}$$

where E, F, G are coefficients of the first fundamental forms, and e, f, g are coefficients of the second fundamental forms. Specifically, E, F, G are defined as

$$E = \frac{\partial \mathbf{s}}{\partial \rho}\cdot\frac{\partial \mathbf{s}}{\partial \rho} = \left[\frac{dR(\rho)}{d\rho}\right]^2 + \left[\frac{dH(\rho)}{d\rho}\right]^2, \tag{S.6}$$

$$F = \frac{\partial \mathbf{s}}{\partial \rho}\cdot\frac{\partial \mathbf{s}}{\partial \varphi} = 0, \tag{S.7}$$

$$G = \frac{\partial \mathbf{s}}{\partial \varphi}\cdot\frac{\partial \mathbf{s}}{\partial \varphi} = R^2(\rho), \tag{S.8}$$

the subscript denotes taking the derivative of the vector over the subscripted variable. E, F, G are indeed the elements of the metric of this surface g_{11}, $g_{12}(g_{21})$, g_{22}, respectively, which implies that given a metric of a curved surface, one

can construct the surface from Eq. (S.6) and (S.8). Furthermore, e, f, g are computed by

$$e = \frac{\partial^2 \mathbf{s}}{\partial \rho^2} \cdot \hat{\mathbf{n}} \tag{S.9}$$

$$= \frac{1}{\sqrt{E(\rho)}} \left[-\frac{dH(\rho)}{d\rho}\frac{d^2R(\rho)}{d\rho^2} + \frac{dR(\rho)}{d\rho}\frac{d^2H(\rho)}{d\rho^2} \right], \tag{S.10}$$

$$f = \frac{\partial^2 \mathbf{s}}{\partial \rho \partial \varphi} \cdot \hat{\mathbf{n}} = 0, \tag{S.11}$$

$$g = \frac{\partial^2 \mathbf{s}}{\partial \varphi^2} \cdot \hat{\mathbf{n}} = \frac{R(\rho)}{\sqrt{E(\rho)}}\frac{dH(\rho)}{d\rho}, \tag{S.12}$$

where

$$\hat{\mathbf{n}} = \frac{\frac{\partial \mathbf{s}}{\partial \rho} \times \frac{\partial \mathbf{s}}{\partial \varphi}}{\left| \frac{\partial \mathbf{s}}{\partial \rho} \times \frac{\partial \mathbf{s}}{\partial \varphi} \right|}$$

$$= \left[-\frac{1}{\sqrt{E(\rho)}}\frac{dH(\rho)}{d\rho}\cos\varphi, -\frac{1}{\sqrt{E(\rho)}}\frac{dH(\rho)}{d\rho}\sin\varphi, \frac{1}{\sqrt{E(\rho)}}\frac{dR(\rho)}{d\rho} \right] \tag{S.13}$$

is the unit normal vector. For simplification, we can use coefficients of the first fundamental forms to represent $R(\rho)$ and $H(\rho)$, and the Gaussian curvature is

$$K(\rho) = E^{-2}G^{-1}\left[E\left(\frac{1}{4}G^{-1}G'^2 - \frac{1}{2}G'' \right) + \frac{1}{4}G'E' \right]. \tag{S.14}$$

For a Schwarzschild black hole, $f = 1 - \frac{r_s}{\rho}$, and the Gaussian curvature is

$$K_{\mathrm{S}}(\rho) = -\frac{r_s}{\rho^3} + \frac{3r_s^2}{4\rho^4}. \tag{S.15}$$

One readily checks that $K_{\mathrm{S}}(\rho) < 0$ for all $\rho > r_s$, i.e., throughout the exterior Schwarzschild region that is realized in our optical analogue. The zero of K_{S} occurs at $\rho = 3r_s/4 < r_s$, inside the event horizon, and is therefore not physically relevant here. The radial dependence of $K_{\mathrm{S}}(\rho)$ is plotted in Fig. S1. The photon-sphere radius $\rho_{\mathrm{PS}} = 3r_s/2$ is indicated by the blue hexagon, illustrating that the Schwarzschild surface is negatively curved for all $\rho > r_s$ in the region realized by our optical analogue.

III. VARIABLE SEPARATION OF THE WAVE EQUATION

In this section, we derive the Eqs. (9) and (10) by performing the variable separation method on the wave equation, which, written in curvilinear coordinates, is

$$\Box\Psi \equiv \frac{1}{\sqrt{g}}\partial_\mu\left(\sqrt{g}\, g^{\mu\nu}\partial_\nu\Psi\right) = 0, \tag{S.16}$$

where $g^{\mu\nu}$ is the element of the inverse matrix of $g_{\mu\nu}$, $g = \det(g_{\mu\nu})$, and Greek indices μ, ν run over the spacetime coordinates (t, ρ, φ). For 2D Schwarzschild surfaces, which are described by the Fermat metric Eq. (3), as they are embedded in 3D flat spacetime, a flat time term should be added to obtain a full (2+1)D spacetime metric as

$$ds^2 = -c^2dt^2 + f^{-2}(\rho)d\rho^2 + \rho^2 f^{-1}(\rho)d\varphi^2. \tag{S.17}$$

Substituting Eq. (S.17) into Eq. (S.16), we have the wave equation

$$\rho^{-1}f^{\frac{3}{2}}(\rho)\frac{\partial}{\partial\rho}\left[\rho f^{\frac{1}{2}}(\rho)\frac{\partial\Psi}{\partial\rho}\right] + \rho^{-2}f(\rho)\frac{\partial^2\Psi}{\partial\varphi^2} - \frac{1}{c^2}\frac{\partial^2\Psi}{\partial t^2} = 0, \tag{S.18}$$

Here a time-relevant term $-c^2dt^2$ is added to the metric Eq. (3), as the surface is embedded in flat spacetime. Assuming variable t can be separated from other variables, we write wave function $\Psi(t,\rho,\varphi) = T(t)Y(\rho,\varphi)$. Substituting it into Eq. (S.18), we have

$$\frac{f^{\frac{3}{2}}(\rho)}{\rho Y(\rho,\varphi)}\frac{\partial}{\partial\rho}\left[\rho f^{\frac{1}{2}}(\rho)\frac{\partial Y(\rho,\varphi)}{\partial\rho}\right] + \frac{f(\rho)}{\rho^2 Y(\rho,\varphi)}\frac{\partial^2 Y(\rho,\varphi)}{\partial\varphi^2} = \frac{1}{T(t)}\frac{1}{c^2}\frac{\partial^2 T(t)}{\partial t^2}. \tag{S.19}$$

As Eq. (S.19) works for arbitrary t,ρ and φ, the only possibility is that both sides equal to a constant, say, $-k^2$. Therefore, one has

$$\frac{\partial^2 T(t)}{\partial t^2} + k^2c^2T(t) = 0 \tag{S.20}$$

$$\frac{f^{\frac{3}{2}}(\rho)}{\rho}\frac{\partial}{\partial\rho}\left[\rho f^{\frac{1}{2}}(\rho)\frac{\partial Y(\rho,\varphi)}{\partial\rho}\right] + \frac{f(\rho)}{\rho^2}\frac{\partial^2 Y(\rho,\varphi)}{\partial\varphi^2} + k^2Y(\rho,\varphi) = 0. \tag{S.21}$$

Eq. (S.20) leads to the oscillating wave solution $T(t) = e^{ickt}$. For Eq. (S.21), due to the rotational symmetry of surfaces of revolution, variable ρ and φ can be separated as $Y(\rho,\varphi) = R(\rho)\Theta(\varphi)$. Substituting it into Eq. (S.21), one has

$$\frac{\rho f^{\frac{1}{2}}(\rho)}{R(\rho)}\frac{d}{d\rho}\left[\rho f^{\frac{1}{2}}(\rho)\frac{dR(\rho)}{d\rho}\right] + \frac{\rho^2}{f(\rho)}k^2 = -\frac{1}{\Theta(\varphi)}\frac{d^2\Theta(\varphi)}{d\varphi^2} \equiv l^2. \tag{S.22}$$

i.e.,

$$\frac{d^2\Theta(\varphi)}{d\varphi^2} + l^2\Theta(\varphi) = 0, \quad \Theta(\varphi) = e^{il\varphi}, \tag{S.23}$$

$$\rho f^{\frac{1}{2}}(\rho)\frac{d}{d\rho}\left[\rho f^{\frac{1}{2}}(\rho)\frac{dR(\rho)}{d\rho}\right] + \left[\frac{\rho^2}{f(\rho)}k^2 - l^2\right]R(\rho) = 0. \tag{S.24}$$

To get the Schrödinger's equation, we apply the ansatz $R(\rho) = \rho^{-\frac{1}{2}}f^{-\frac{1}{4}}(\rho)\psi(\rho)$. After tedious algebraic calculation, one has

$$\frac{d^2\psi(\rho)}{d\rho^2} + \frac{\rho^2}{(\rho - r_s)^2}\left[k^2 - \left(l^2 + \frac{1}{2}\right)\frac{\rho - r_s}{\rho^3} + \frac{3}{16}\frac{(2\rho - r_s)^2}{\rho^4}\right]\psi(\rho) = 0, \tag{S.25}$$

which corresponds to Eqs. (9) and (10) in the main text.

To illustrate the structure of the photon-sphere modes obtained from the Schrödinger-like equation above, we show in Fig. S2 four different PS orders. Panel (a) displays the effective potential $V_{\text{eff}}(\rho)$ used in the main text, together with the range of k^2 values (orange dashed lines) where PS modes exist. Panel (b) shows the corresponding function $Q(\rho)$ entering Eq. (9) of the main text, while panel (c) presents the determinant of the coefficient matrix whose zeros give the allowed PS eigenvalues. Panels (d)–(f) plot the resulting modes as radial intensity profiles along ρ and as intensity distributions on the Schwarzschild surface and on the conformally transformed 2D plane.

To complement the photon-sphere modes discussed above, we also illustrate the structure of whispering-gallery modes (WGMs) supported by the Schwarzschild cavity. Figure S3 shows five representative WGMs obtained from the theoretical model, corresponding to different radial orders within the branches plotted in Fig. 2(c) of the main text. As the radial order increases, the field becomes more tightly confined near the outer boundary of the surface.

IV. MEASUREMENT OF THE REFRACTIVE INDEX

To obtain the group refractive index of the 3D laser microcavities, we 3D-print a cuboid microlaser of side length a=100 μm in the same dye-doped resin. A SEM image of the fabricated structure is shown in Fig. S4(a). Top view of the cuboid laser under white light, with focus on the upper surface is shown in the inset of Fig. S4(b). The cuboid microlaser is then pumped with a square-shaped pump profile of 120 μm x 120 μm dimension. When the pump power is sufficiently high, we observe an emission spectrum with regularly spaced peaks as shown in Fig. S4(b).

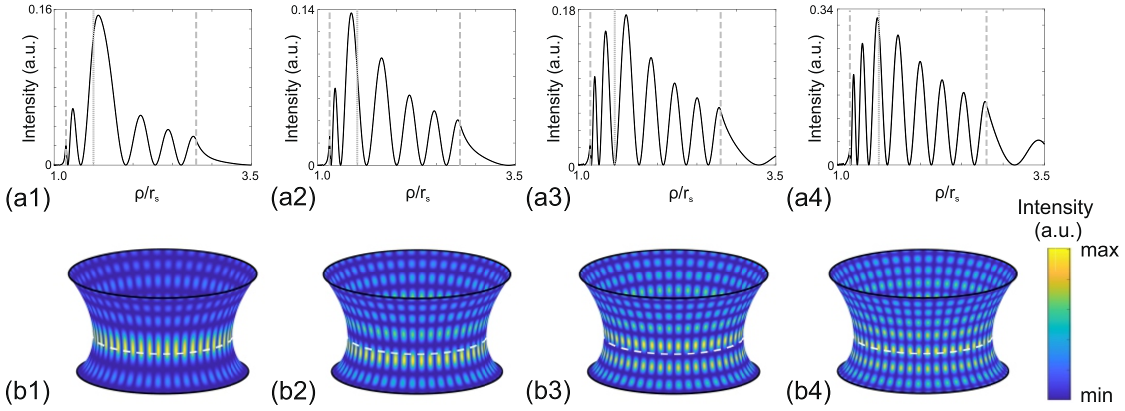

FIG. S2: Different orders of photon sphere modes. (a) Effective potential Schwarzschild surface as shown in the main text, whose range of square of eigen-wave number k^2 is limited by the orange dot lines. (b) Term $Q(\rho)$ of different regions. (c) Determinant of coefficient matrix, whose zeros correspond to photon sphere modes, denoted by orange stars. Intensity distribution of these four photon sphere modes along ρ direction (d), on Schwarzschild surface (e) and on 2D transformed plane (f). The boundaries of Schwarzschild surface are denoted by gray dashed lines (d) and black (e)/white (f) solid lines, while the photon sphere is denoted by gray dot lines (d) and white dashed lines (e,f).

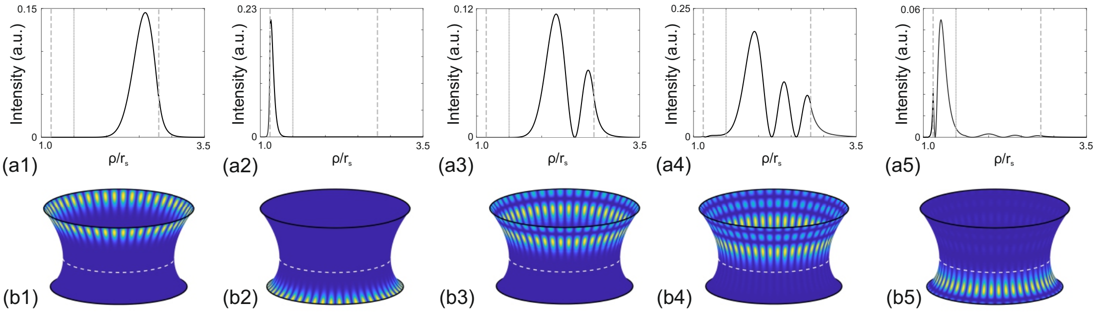

FIG. S3: Five representative whispering-gallery modes of the Schwarzschild cavity. Each panel shows the intensity distribution $|\Psi|^2$ on the Schwarzschild surface for a mode belonging to one of the WGM branches in Fig. 2(c) of the main text.

The comb-like spectrum corresponds to a diamond periodic orbit excited in the cuboid laser [2]. The inset of Fig. S4(c) shows the top view of the cuboid laser when pumped with square-shaped pump. Fig. S4(c) shows the Fourier transform of the emission spectrum, which consists of regularly spaced harmonics. Its first peak at nL=440 μm gives the optical path length corresponding to the diamond orbit, where $L = 2\sqrt{2}a$ is the geometrical length of the orbit and n is the group refractive index of the cuboid laser. For the given optical path length, the group refractive index is calculated as n=1.56. Appendix V of Ref. [3] discuss the precision of this measurement.

V. EXPERIMENTAL OBSERVATION OF A SECOND-ORDER WHISPERING-GALLERY MODE

To confirm experimentally the existence of higher-order whispering-gallery modes in the laser microcavity, we selectively pump the cavity at the location predicted for the second-order WGM by the theoretical analysis. The top view of the structure with the pump stripe placed at this position is shown in the inset of Fig. S5(a). When the pump power exceeds threshold, the emission spectrum exhibits a comb-like structure, as presented in Fig. S5(a).

To identify the lasing mode responsible for this comb, we perform a Lomb–Scargle analysis of the spectrum. The corresponding periodogram, shown in Fig. S5(b), displays regularly spaced peaks. The position of the first peak gives the optical path length $nL_3 = 266.4$ μm. Assuming that the lasing mode is confined on a circular orbit, this corresponds to an effective diameter of 54.4 μm, in good agreement with the second-order WGM location inferred from the theoretical model.

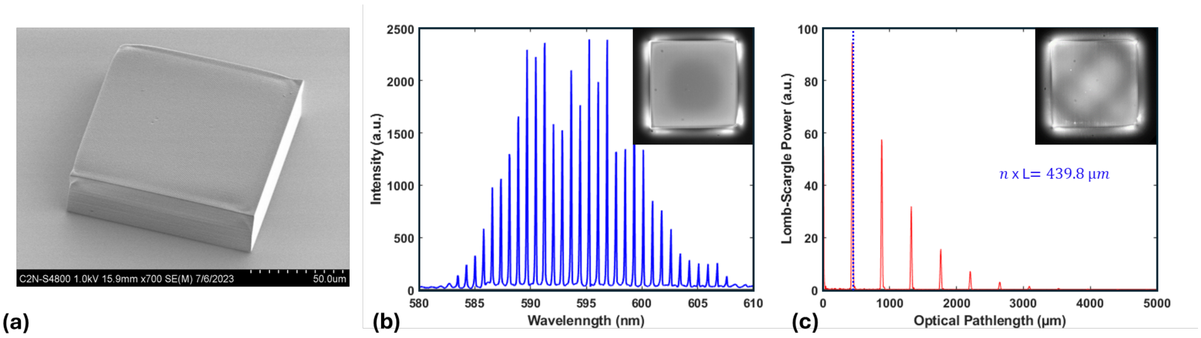

FIG. S4: (a) SEM image of a cuboid microlaser. (b) Emission spectrum of a cuboid microlaser when pumped with a square-shaped pump of dimension 120 μm x 120 μm. Inset of the figure shows the top view of cuboid microlaser under white light illumination, with focus on the upper surface. (c) Fourier transform of the emission spectrum in (b). Inset of the figure shows the top view of the cuboid microlaser when pumped with laser.

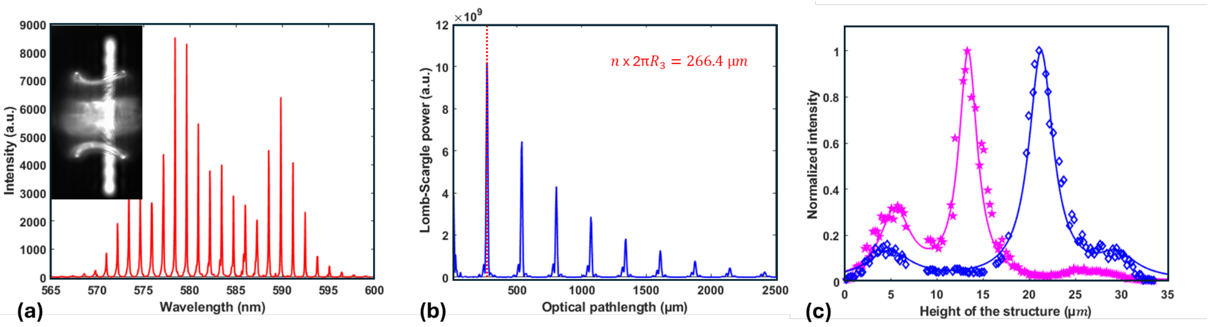

FIG. S5: (a) Emission spectrum of the laser microcavity when selectively pumped at the location of the second-order whispering-gallery mode. The inset shows the top view of the structure with the pump stripe positioned at this location. (b) Lomb–Scargle analysis of the spectrum in (a). The first peak yields the optical path length $nL_3 = 266.4$ μm, consistent with a second-order WGM confined on a circular orbit of effective diameter 54.4 μm.

[1] C. Xu, I. Dana, L. G. Wang, P. Sebbah, Light chaotic dynamics in the transformation from curved to flat surfaces, Proc. Natl. Acad. Sci. USA **119**, e2112052119 (2022).

[2] V. Chen, N. Sobeshchuk, C. Lafargue, E. Mansfield, J. Yom, L. Johnstone, R. Lucas J. Hales, S. Bittner, S. Charpignon, D. Ulbricht, J. Lautru, I. Denisyuk, J. Zyss, J. Perry, and M. Lebental, *Three-dimensional organic microlasers with low lasing thresholds fabricated by multiphoton and UV lithography*, Optics Express, **22,** 12316 (2014).

[3] Y. Song, Y. Monceaux, S. Bittner, K. Chao, H. M. Reynoso de la Cruz, C. Lafargue, D. Decanini, B. Dietz, J. Zyss, A. Grigis, X. Checoury, and M. Lebental, *Möbius Strip Microlasers: A Testbed for Non-Euclidean Photonics*, Phys. Rev. Lett. , **127**, 203901 (2021).
